# Comparing Emotional Development in Persons With Intellectual Disability With and Without Autism Spectrum Disorder

**DOI:** 10.1111/jir.13251

**Published:** 2025-05-14

**Authors:** Hauke Hermann, Annemieke M. Witte, Anna Pöhlmann, Paula S. Sterkenburg, Tanja Sappok

**Affiliations:** ^1^ Bielefeld University, Medical School and University Medical Center OWL, Mara Hospital, University Clinic for People With Neurodevelopmental Disorders Bielefeld Germany; ^2^ Charité – Universitätsmedizin Berlin, corporate member of Freie Universität Berlin and Humboldt‐Universität zu Berlin Berlin Germany; ^3^ Clinical Child and Family Studies Vrije Universiteit Amsterdam Amsterdam The Netherlands; ^4^ Department for Assessment and Treatment Bartiméus Doorn The Netherlands

**Keywords:** autism, emotional development, intellectual disability, mental health, neurodevelopmental disorders, scale of emotional development‐short

## Abstract

**Background:**

Intellectual disability (ID) often co‐occurs with autism spectrum disorder (ASD). To better understand the needs of persons with ID/ASD, level of emotional development (ED) can be determined with the Scale of Emotional Development‐Short (SED‐S). This preregistered study examined differences in ED by comparing total, domain, and item scores between people with ID/ASD and people with ID.

**Methods:**

One hundred seventy‐four participants with ID/ASD were matched to 174 participants with ID only. Informants reported on the SED‐S, which includes 200 yes‐no items grouped into eight domains, with each domain including five stages of ED.

**Results:**

The ID/ASD group showed lower total scores (*M* = 2.19, *SD* = 0.97) compared with the ID group (*M* = 2.86, *SD* = 1.11). They also showed lower scores in all eight domains. When groups were compared based on total scores, people with ID/ASD in SED‐S 2 scored lower in the domain *Affect*, while those in SED‐S 3 scored lower in the domains *Affect*, *Communication*, and *Peers* compared with people with ID in the same stage. People with ID/ASD in SED‐S 4 scored higher in the domain *Peers* compared with people with ID in the same stage. There was an uneven distribution of ‘yes’ responses, significant differences in ‘yes’ responses to 27 items, and a lower mean frequency of ‘yes’ responses from people with ID/ASD.

**Conclusions:**

Although this study was largely exploratory and warrants replication, results provide an important next step towards a better understanding of the emotional needs and behaviours of people with ID/ASD.

## 1 Introduction

The co‐occurrence of intellectual disability (ID) and autism spectrum disorder (ASD) is a topic of scientific and clinical interest due to its impact on assessment, prognosis, and treatment response. ID is characterized by impairments in intellectual functioning (e.g., learning and problem solving) and problems with adaptive behaviour (e.g., social and practical skills), while ASD is characterized by impairments in the ability to initiate and continue social communication and interaction, and by restricted, repetitive, inflexible behaviours and/or atypical interests (ICD‐11; World Health Organization 2018). Both disorders manifest during the developmental period and are life‐long conditions. People with ID, who are more likely to experience co‐occurring physical (Liao et al. 2021) and mental health problems (Mazza et al. 2020), are at even greater risk for adverse health outcomes when diagnosed with a co‐occurring ASD (Bakken et al. 2010; Cervantes and Matson 2015; Dunn et al. 2020). Furthermore, challenging behaviours, such as self‐injury, aggression, and stereotypy, are more frequently displayed by people with ID and ASD compared with people with ID only (Álvarez‐Couto et al. 2024; Esteves et al. 2021; O'Dwyer et al. 2018). Assessing the level of emotional development (ED)—the extent to which individuals can perceive, express and regulate different emotions—can help to better distinguish between mental health problems and behavioural phenomena (Hermann et al. 2022). The aim of this preregistered study was to examine differences in ED between people with ID and ASD and people with ID only.

In the early 2000s, Došen (2005a, 2005b) presented the developmental‐dynamic approach, which not only integrates developmental principles from theories on cognitive (Piaget 1954), psychodynamic (Mahler et al. 1975), social (Erikson 1959), self/ego (Stern 1985) and attachment (Bowlby 1969) development but also scientific findings on the maturation and neurotomy of socio‐emotional brain circuits (Casey et al. 2000; Huttenlocher 1984; Johnson et al. 2005; LeDoux 2000; Nelson and Cicchetti 1995). More specifically, Došen's approach describes five consecutive stages of ED that run parallel with the normative trajectories observed in typically developing children aged 0 to 12 years old. In each stage, individuals posit specific emotional needs, motivations, and coping mechanisms that affect adaptive and maladaptive behaviours in everyday life. People with ID can pass through the five stages of ED but incomplete or delayed development is commonly observed (e.g., Flachsmeyer et al. 2023; Hermann et al. 2022; Meinecke et al. 2024). The assessment of ED seems important for interpreting complex behaviours in light of underlying emotional processes. Indeed, Barrett et al. (2024) showed that the assessment of ED was associated with fewer psychotropic and antipsychotic medication prescriptions, not only in individuals with ID displaying challenging behaviours but also in individuals with ASD (Barrett et al. 2024).

Currently, several instruments have been introduced to measure ED, including the Scheme for Appraisal of Emotional Development (SAED; Došen 2005a, 2005b), the Scale for Emotional Development‐Revised (SED‐R; Claes and Verduyn 2011) and the SED‐R second revision (SED‐R^2^; Morisse and Došen 2017). Together these instruments laid the foundation for the development of the Scale of Emotional Development‐Short (SED‐S; Sappok et al. 2016). The SED‐S is a semistructured informant‐based interview which can be used in the diagnostic and scientific field. The SED‐S assigns a developmental stage (1 to 5) to each of the eight domains and subsequently provides an overall stage of ED. The SED‐S is a standardized (Sappok, Došen, et al. 2020) and validated instrument (Sappok et al. 2016, 2019; Sappok, Došen, et al. 2020). The SED‐S demonstrated consistently good psychometric properties in both samples of children (Sterkenburg et al. 2021) and adults with ID (Flachsmeyer et al. 2023; Hermann et al. 2024; Meinecke et al. 2024).

It may not be surprising that people with ID and ASD are expected to show lower levels of ED than people with ID only. Leo Kanner (1943) described autism as a condition where people are unable to ‘form affective contact with people’ (250). The current diagnostic criteria for ASD include deficits in emotional competencies (American Psychiatric Association 2022; World Health Organization 2018, see also Begeer et al. 2008). However, it is worth noting that ASD is a spectrum disorder, with some individuals showing milder deficits in their emotional competencies and others facing more significant impairments (World Health Organization 2018).

Currently, few studies have examined differences in ED between people with ID and ASD and those with ID only. A first study that used the Scheme of Appraisal of Emotional Development (SEAD) reported lower overall levels of ED in people with ID and ASD (mean reference age: 18–35 months; stage of individuation) than in people with ID only (mean reference age: 3–7 years old; stage of identification) (Sappok et al. 2013). Also, people with ID and ASD showed lower levels of ED in 9 of the 10 SEAD domains. When stratified by the level of ID, differences in the overall level of ED remained, but results varied for the different domain levels. In a later study with the SED‐S, people with ID and ASD displayed lower levels of ED overall (mean reference age: 7–18 months; stage of socialization) than people with ID only (mean reference age 19–36; stage of individuation), as well as in all SED‐S domains, with the most pronounced differences in the domains ‘Relating to Peers’, ‘Engaging with the Material World’ and ‘Differentiating Emotions’(Sappok, Heinrich, and Böhm 2020). When stratified according to severity of ID, people with mild/moderate ID and ASD showed lower levels of ED in three domains: ‘Differentiating Emotions’, ‘Relating to Peers’ and ‘Communicating with Others’, and people with severe/profound ID and ASD showed lower levels of ED in five domains: ‘Differentiating Emotions’, ‘Relating to Peers’ and ‘Dealing with Change/Object Permanence’, ‘Engaging with the Material World’ and ‘Regulating Affect’ compared to people with similar severity of ID (Sappok, Heinrich, and Böhm 2020). These findings underscore the importance for accounting for severity of ID to understand differences in ED. Finally, Sterkenburg et al. (2021) included a sample of children with ID and reported lower levels of ED overall and in the domains ‘Differentiating Emotions’, ‘Relating to Peers’, ‘Communicating with Others’, ‘Regulating Affect’ and ‘Relating to Significant Others’ among the group where children had an additional ASD diagnosis. Notably, groups did not differ in age or severity of ID (Sterkenburg et al. 2021).

It can be concluded that the aforementioned studies, although few in number and in need of replication, consistently reported diminished levels of ED in people with ID and ASD. The present study will add to this previous research by examining differences in ED, not only on overall and domain level but also on item level, which will result in a more detailed and comprehensive understanding of ED in people with ID with and without ASD. Additionally, findings may help to broaden general understanding about ASD. This study adopts a different statistical approach than previous studies, as a propensity matching procedure will be used to adjust for differences in ID and gender, rather than conducting analyses in subgroups or regression analyses (Sappok et al. 2013; Sappok, Heinrich, and Böhm 2020). Propensity score matching enables a direct comparison between groups without relying on assumptions or specifications associated with statistical models. Furthermore, it allows group comparisons across multiple levels, including the overall level, domain level and item level.

In sum, the aim of this preregistered study (https://osf.io/8n7zk) is to examine differences in ED between people with ID and ASD and people with ID only. More specifically, we examined differences in ED with respect to (1) SED‐S total scores, (2) SED‐S domain scores and (3) SED‐S item scores. Based on previous findings (Sappok et al. 2013; Sappok, Heinrich, and Böhm 2020), it is expected that people with ID and ASD show lower total scores of ED. We did not formulate hypotheses for the domain outcomes due to limited evidence and different methodological approaches. We also did not formulate hypotheses for the item‐analyses as they were exploratory in nature.

## 2 Method

### 2.1 Participants

The sample consisted of 174 participants with ID/ASD who were matched to a group of 174 participants with ID only. The latter group was drawn from a larger sample of 435 participants with ID only. Table 1 provides an overview of participant characteristics before matching, presented with absolute and relative frequencies. Participants were recruited from hospitals, residential locations, day‐care centres and supported‐employment services in Belgium, Germany and the Netherlands. More specifically, the following organizations participated: Tordale in Torhout (Belgium), the Evangelisches Krankenhaus Königin Elisabeth Herzberge in Berlin (Germany), St. Lukas‐Klink in Liebenau (Germany), Cordaan in Amsterdam (the Netherlands), ORO in Helmond (the Netherlands), Bartiméus in Doorn (the Netherlands) and De Twentse Zorgcentra in Losser (the Netherlands). Data were collected between March 2016 and November 2020. The study was preregistered on the Open Science Framework (see: https://osf.io/8n7zk). Exclusion criteria included a diagnosis of dementia or an amnestic disorder and incomplete datasets. Participants and/or legal guardians provided written informed consent prior to the start of the study. In some cases, informed consent was not obtained since the Landeskrankenhausgesetz (State Hospital Act) states that this is not necessary if the analyses are retrospective in nature and anonymity is preserved, which was the case. This study was performed according to the principles of the Declaration of Helsinki (World Medical Association n.d.). Ethical approval was obtained for each participating organization (see also Hermann et al. 2024 for more details about the ethical approval).

**TABLE 1:** Description of the group comparison sample prior to matching, including the ID/ASD group (*n* = 174) and the ID group (*n* = 435).

	Characteristics	ID/ASD *n* (%)	ID *n* (%)
Descriptives	Gender male	116 (66.7)	247 (56.8)
	Gender female	58 (33.3)	188 (43.2)
	Mean age (years)	37.80	36.20
Level of ID	Mild (ICD‐10: F70)	31 (17.8)	133 (30.6)
	Moderate (ICD‐10: F71)	51 (29.3)	165 (37.9)
	Severe (ICD‐10: F72)	77 (44.3)	98 (22.5)
	Profound (ICD‐10: F73)	15 (8.6)	39 (9.0)
Mental illness	Psychoactive substances (F1x.x)	1 (0.6)	8 (1.8)
	Schizophrenia (F2x.x)	20 (11.5)	41 (9.4)
	Affective disorders (F3x.x)	20 (11.5)	55 (12.7)
	Neurotic disorders (F4x.x)	7 (4.0)	51 (11.7)
	Personality disorders (F6x.x)	2 (1.1)	14 (3.2)

*Note:* Persons may be diagnosed with multiple disorders. The International Classification of Diseases and Related Health Problems, 10th edition (ICD‐10) was used for diagnosis. Fxx.x refers to codes and categories from the ICD‐10.

Abbreviation: ID, intellectual disability.

### 2.2 Measurements

#### 2.2.1 Emotional Development

Emotional development (ED) was measured with the Scale of Emotional Development‐Short (SED‐S; Sappok et al. 2016), a semistructured interview consisting of 200 items. At least two informants (i.e., family members or close caregivers) indicated in a binary manner (‘yes’ or ‘no’) whether the items described behaviours that were exhibited by the participant in the 2–12 weeks preceding the interview. Items are grouped into eight domains: ‘Relating to his/her Own Body’ (*Body*), ‘Relating to Significant Others’ (*Others*), ‘Dealing with Change/Object Permanence’ (*Object*), ‘Differentiating Emotions’ (*Emotions*), ‘Relating to Peers’ (*Peers*), ‘Engaging with the Material World’ (*Material*), ‘Communicating with Others’ (*Others*) and ‘Regulating Affect’ (*Affect*) (see also Tarasova et al. 2022 for an extension of the scale). Each domain includes 25 items, with five items corresponding to one of the five stages of ED: Adaption (0–6 months), Socialization (6–18 months), First Individuation (18–36 months), Identification (3–7 years) and Reality Awareness (7–12 years). Level of ED in each domain is determined by selecting the stage with the highest number of ‘yes’ responses. If stages received an equal number of ‘yes’ responses, the lower domain stage was selected. Overall level of ED is determined by ranking the domain stages from lowest to highest and then selecting the fourth lowest domain stage (Sappok et al. 2016). The SED‐S was administered by trained professionals, mostly psychologists, and took approximately 30–60 min to complete.

#### 2.2.2 Autism Spectrum Disorder

The autism diagnosis was made by independent clinicians using the diagnostic criteria of the ICD‐10. Diagnoses of childhood autism (F84.0) and atypical autism (F84.1) were grouped together in the term ‘Autism Spectrum Disorder’ (ASD).

### 2.3 Data‐Analysis Plan

We conducted propensity score matching to adjust for differences between the ID/ASD and ID group in gender and severity of ID. This approach facilitates a direct comparison of the two groups without the need to meet assumptions or specifications from statistical models. First, differences in SED‐S total scores were examined using a Brunner‐Munzel test. Second, differences in SED‐S domain scores were analysed using three steps: (1) nonparametric multiple contrast tests for repeated measures, which account for multiple testing (Rubarth et al. 2022), were performed to explore heterogeneity in the domain scores of the ID/ASD group, (2) Brunner‐Munzel tests were conducted to explore differences in the domain scores between the ID/ASD and ID group and (3) in case of significant differences, additional Brunner–Munzel were preformed to explore differences in domain scores per assigned SED‐S stage. Finally, descriptive statistics and Chi‐Squared tests were used to examine differences with respect to number of yes‐answers on item level. Given the exploratory nature of the study, *p*‐values were interpreted as exploratory rather than confirmatory.

## 3 Results

### 3.1 Preliminary Analyses

Statistical analyses prior to matching, based on absolute and relative frequencies, showed that there were relevant differences between the ID/ASD (*n* = 174) and ID group (*n* = 435). That is, more men were included in the ID/ASD group (66.7%) compared with the ID group (56.8%) (see also Table 1). Furthermore, more people with severe to profound ID were included in the ID/ASD group compared with the ID group. Differences were adjusted for using the propensity score matching method, resulting in two comparable study groups, that is, the ID/ASD group (*n* = 174) and the ID group (*n* = 174).

### 3.2 Differences Between the ID/ASD and ID Group in SED‐S Total Scores

**FIGURE 1:**
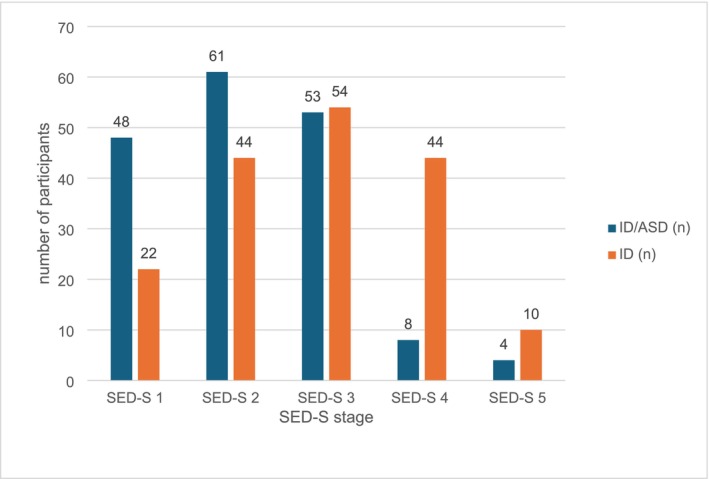
Distribution of SED‐S total score for the ID/ASD and ID group.

Figure 1 shows the distribution of the SED‐S total score for the ID/ASD (orange) and ID group (blue). Findings indicate that the ID/ASD group was predominantly rated with SED‐S stages 1 to 3, while the ID group was predominantly represented by SED‐S stages 2 to 4. With respect to the highest developmental stages, eight individuals in the ID/ASD group were assigned to SED‐S 4, and four individuals were assigned to the SED‐S 5, compared with 44 and 10 individuals, respectively, in the ID group. The average SED‐S total score for people in the ID/ASD group was 2.19 (median = 2.0, IQR = 1–3, *SD* = 0.97) and for people in the ID group 2.86 (median = 3, IQR = 2–4, *SD* = 1.11). Results of the Brunner–Munzel test indicated that the ID/ASD group showed lower SED‐S total scores than the ID group, estimated probability (θ) = 0.33, 95% CI [0.27, 0.38], *p* < 0.001.

### 3.3 Domain Scores

#### 3.3.1 Heterogeneous ED Profiles

**FIGURE 2:**
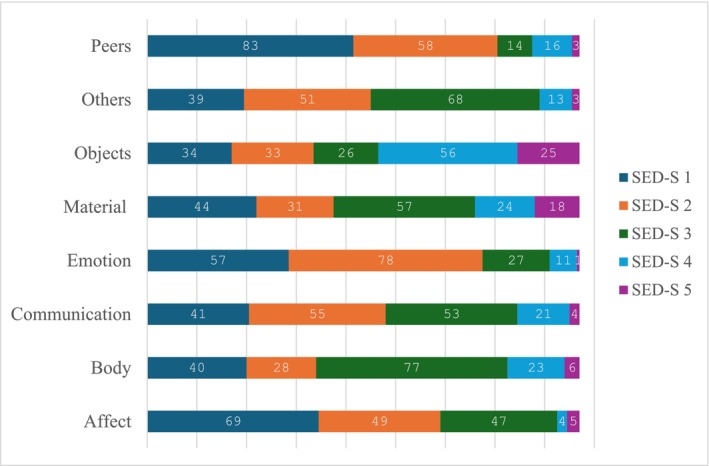
Distribution of SED‐S scores across the domains for the ID/ASD group (*n* = 174). *Note:* Example for interpretation: 83 persons with ID/ASD were assigned to SED‐S level 1 in the domain Peers.

Figure 2 presents the distribution of the domain scores for the ID/ASD group. For the ID/ASD group we performed nonparametric multiple contrast tests for repeated measures to explore heterogeneity in the domain scores. That is, scores in a specific domain were compared with scores in the other domains, resulting in 35 comparisons (i.e., seven comparisons per domain). Results indicated differences for nearly all comparisons, suggesting variability in functioning across domains. Only three domain comparisons did not differ: *Affect* vs. *Emotion* (*p* = 0.74), *Body* vs. *Material* (*p* = 0.88), and *Communication* vs. *Others* (*p* = 0.91). All seven comparisons for *Peers* indicated lower scores in the *Peers* domain, six comparisons for *Emotion* indicated lower scores in the *Emotion* domain, and five and four comparisons for *Affect* and *Communication* indicated lower scores in these domains, respectively, as compared with the scores in the other domains. All seven comparisons for *Object* revealed higher scores in the *Object* domain, six comparisons for *Material* revealed higher scores in the *Material* domain, and five and four comparisons for the *Body* and *Other* revealed higher scores in these domains, respectively, as compared with the scores in the other domains. A more detailed description of the results is presented in Table S1.

#### 3.3.2 Differences Between People With and Without ASD on Domain Level

**FIGURE 3:**
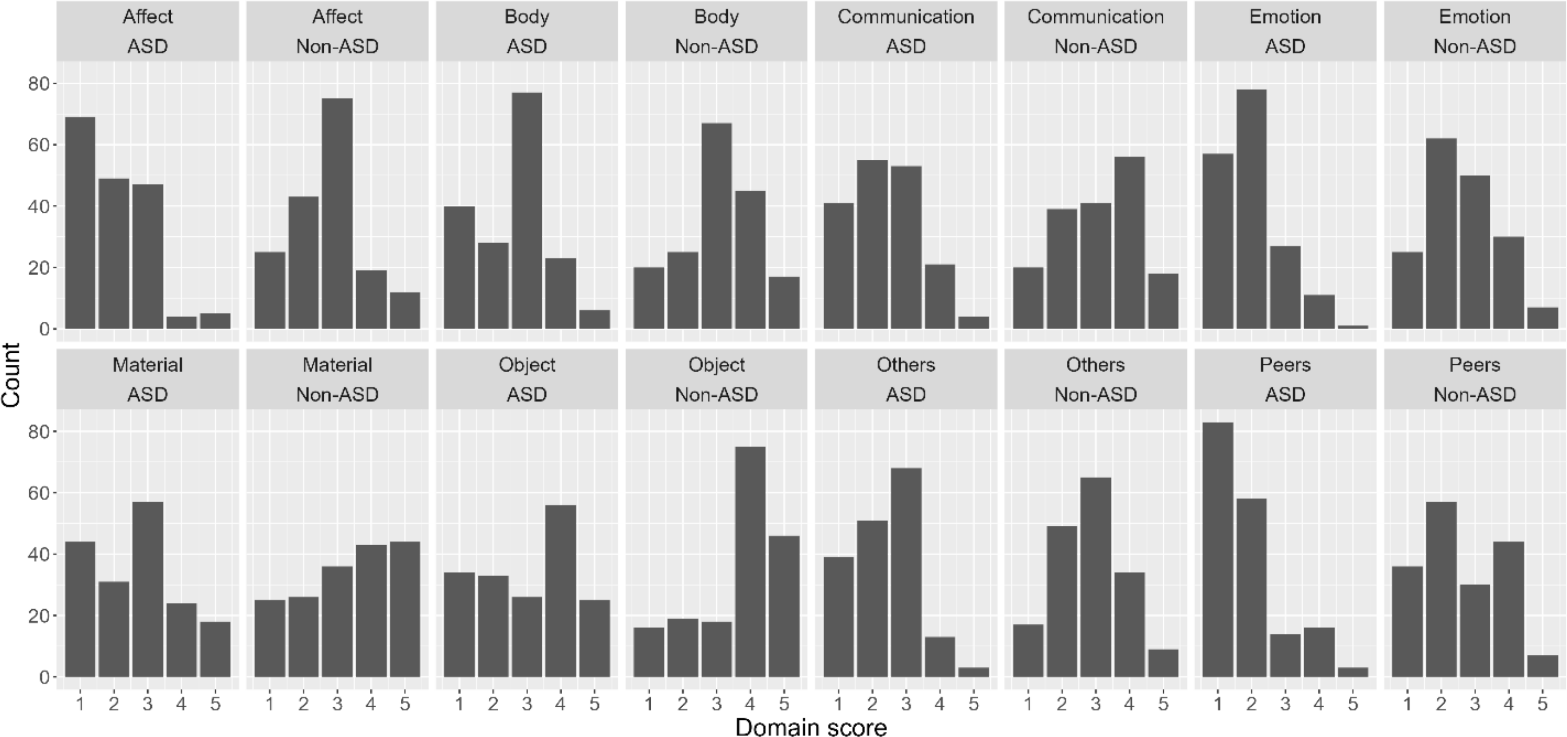
Descriptive representation of the distribution of the SEDS‐S scores for each of the eight domains, presented side by side for the ID/ASD and ID group.

Figure 3 shows the distribution of the SED‐S scores across the domains for both the ID/ASD and ID group (note that for the ID/ASD group the numbers correspond to Figure 2). A first visual inspection showed lower SED‐S scores for the ID/ASD group in the domains *Affect*, *Emotion* and *Peers* compared with the ID group. Brunner–Munzel tests revealed that the ID/ASD group showed lower SED‐S scores in all eight domains compared with the ID group (*p* < 0.01 in every domain), indicating lower levels of ED in all developmental domains.

#### 3.3.3 Differences Between People With and Without ASD on Domain Level for Each SED‐S Stage

Given that direct comparisons revealed lower scores in all eight domains for the ID/ASD group compared with the ID group, we performed additional Brunner–Munzel tests to determine whether differences in domain scores remained or disappeared when groups were stratified according to SED‐S total score (see Figure 1 and Table 2). In stage 1, no significant differences in domain scores were found between people with ID with and without ASD. In stage 2, the ID/ASD group showed lower scores in the domain *Affect* than the ID group. In stage 3, the ID/ASD group showed lower scores in the domains *Affect*, *Communication* and *Peers* than the ID group. In stage 4, the ID/ASD group showed higher scores in the domain *Peers* than the ID group. Due to the small number of cases, group comparisons were not conducted for SED‐S 5.

**TABLE 2:** Comparison of domain scores for the ID/ASD and ID group for each SED‐S score.

SED‐S score	*n*	Domain	Estimate	*p*
SED‐S 1	48 (ID/ASD) and 22 (ID)	Affect	0.46	0.44
		Body	0.48	0.78
		Communication	0.48	0.78
		Emotion	0.41	0.12
		Material	0.53	0.62
		Object	0.47	0.71
		Others	0.44	0.36
		Peers	0.51	0.77
SED‐S 2	61 (ID/ASD) and 44 (ID)	**Affect**	**0.36**	0.01*
		Body	0.56	0.25
		Communication	0.48	0.70
		Emotion	0.48	0.65
		Material	0.60	0.06
		Object	0.48	0.77
		Others	0.49	0.84
		Peers	0.42	0.10
SED‐S 3	53 (ID/ASD) and 54 (ID)	**Affect**	**0.40**	0.02*
		Body	0.51	0.77
		**Communication**	**0.40**	0.03*
		Emotion	0.42	0.09
		Material	0.40	0.06
		Object	0.49	0.83
		Others	0.57	0.10
		**Peers**	**0.32**	< 0.01**
SED‐S‐4	8 (ID/ASD) and 44 (ID)	Affect	0.65	0.17
		Body	0.41	0.45
		Communication	0.48	0.78
		Emotion	0.48	0.85
		Material	0.48	0.82
		Object	0.42	0.50
		Others	0.63	0.25
		**Peers**	**0.70**	< 0.01*

*Note:* Estimate > 0.5 means there is a trend towards higher scores in the ID/ASD group, conversely estimate < 0.5 means there is an indication of lower scores in the ID/ASD group. Significant differences are highlighted in bold.

*p < 0.05, **p < 0.01.

### 3.4 Differences in Item Level Scores Between the ASD and ID Group

For stage 1, the ID/ASD group had relatively more ‘yes’ answers to 22 items, while the ID group had relatively more ‘yes’ answers to 18 items. For stage 2, the ID/ASD group had relatively more ‘yes’ answers to six items, while the ID group had relatively more ‘yes’ answers to 31 items; there were three items with similar percentages. For stage 3, the ID/ASD group had relatively more ‘yes’ answers to six items, while the ID group had relatively more ‘yes’ answers to 34 items. For stage 4 and 5, the ID/ASD group had more ‘yes’ answers to 13 items and 8 items, respectively, while the ID group had more ‘yes’ answers to 27 items and 26 items. For SED‐S 5, there were six items with similar percentages. Significant differences emerged for 27 out of 200 items (see Table 3). More specifically, SED‐S 1 included two items that scored higher in the ID/ASD group (*Peers; n* = 1, *Affect; n* = 1). SED‐S 2 included nine items (Body; *n* = 2, *Object*; *n* = 1; Emotion; *n* = 2, *Peers*; *n* = 2, *Materials; n* = 2), SED‐S 3 included 12 items (*Body; n* = 2, *Emotion*; *n* = 3, *Peers; n* = 3, *Material*; *n* = 1, *Communication*; *n* = 1, *Affect; n* = 2), SED‐S 4 included two items (*Materials*; *n* = 2) and SED‐S 5 included two items (*Peers; n* = 2) for which the number of yes‐responses was higher in the ID group (see Tables S2–S6 for more details). Finally, descriptive analyses indicated that the mean score for the frequency of ‘yes’ answers was higher for the ID group (*M* = 10.79, *SD* = 9.41) compared with the ID/ASD group (*M* = 4.11, *SD* = 5.84).

**TABLE 3:** Items with significant differences in the number of yes responses between the ID/ASD and ID group.

SED‐S	Item description	Domain and item number	Overall	ID/ASD *n* (%)	ID group *n* (%)	*p*
1	Shows no interest in peers	Peers 1_1	48	38 (79)	10 (45)	0.005
1	Displays auto‐aggressive behaviour	Affect 1_5	57	43 (90)	14 (64)	0.010
2	Uses his/her body as an instrument to explore the immediate environment (e.g., switching the light on and off repeatedly etc.)	Body 2_2	52	25 (41)	27 (61)	0.039
2	Body‐focused attention (e.g., during bathing, brushing hair) leads to pleasurable interactions	Body 2_5	49	22 (36)	27 (61)	0.010
2	Enjoys playing peek‐a‐boo and hide‐and‐seek	Object 2_4	23	8 (13)	15 (34)	0.010
2	Enjoys activities with significant others	Emotion 2_2	81	42 (69)	39 (89)	0.017
2	Emotional states can be influenced by attention from caregivers	Emotion 2_4	80	42 (69)	38 (86)	0.038
2	Is curious about peers and interacts with them briefly	Peers 2_2	47	22 (36)	25 (57)	0.035
2	Engages with peers when authority figures are present	Peers 2_5	34	15 (25)	19 (43)	0.045
2	Enjoys interactions that involve repeatedly throwing, dropping and handing objects back and forth	Material 2_3	21	7 (11)	14 (32)	0.010
2	Reaches for things he/she can see or hear	Material 2_5	57	27 (44)	30 (68)	0.015
3	Seeks help from others or utilizes objects to overcome physical limitations (uses a chair to reach the cookie jar, for example)	Body 3_2	87	37 (70)	50 (93)	0.003
3	Uses language (possibly supported by gestures) to communicate	Body 3_4	99	46 (87)	53 (98)	0.026
3	Wants caregivers to him−/herself, shows jealousy	Emotion 3_2	43	16 (30)	27 (50)	0.037
3	Is able to name own basic feelings (e.g., anger, sadness, fear, happiness)	Emotion 3_4	58	22 (42)	36 (67)	0.009
3	Wants to be the center of attention	Emotion 3_5	46	16 (30)	30 (56)	0.008
3	Tries to impose his/her will on peers	Peers 3_1	60	22 (42)	38 (70)	0.003
3	Shows no regard for what peers want	Peers 3_3	57	22 (42)	35 (65)	0.016
3	Bosses peers around	Peers 3_5	47	16 (30)	31 (57)	0.005
3	Does activities in which imitation plays a role	Material 3_5	20	5 (9)	15 (28)	0.015
3	Uses ‘bad’ words to provoke reactions from others	Communication 3_4	31	10 (19)	21 (39)	0.022
3	Frustration finds expression in physical restlessness, temper tantrums and stubbornly oppositional behaviour	Affect 3_3	89	39 (74)	50 (93)	0.009
3	Is rarely able to talk about the causes and effects of his/her aggressive behaviour	Affect 3_5	67	27 (51)	40 (74)	0.013
4	Approaches peers by play and shared activities	Material 4_1	34	1 (12)	33 (75)	0.001
4	Follows simple rules (like waiting his/her turn) when playing games	Material 4_5	51	7 (88)	44 (100)	0.018
5	Shows loyalty to friends	Peers 5_4	12	2 (50)	10 (100)	0.016
5	Consults with peers to find solutions to conflicts/problems	Peers 5_5	8	0 (0)	8 (80)	0.006

## 4 Discussion

This preregistered study used a propensity score matching approach to examine differences in ED between people with ID and ASD and those with ID only. Specifically, groups were compared on the total, domain and item scores of the SED‐S. In line with our expectations and with previous research (Sappok et al. 2013; Sappok, Heinrich, and Böhm 2020), people with ID and ASD showed lower total scores of ED than those with ID only, suggesting that ASD contributes to additional challenges in ED. Furthermore, people with ID and ASD showed lower scores in all eight SED‐S domains. When groups were stratified according to the SED‐S total score, people with ID and ASD in SED‐S 2 scored lower in the domain *Affect*, while people with ID and ASD in SED‐S 3 scored lower in the domains *Affect*, *Communication* and *Peers* than those with ID only in the respective SED‐S stages. Surprisingly, people with ID and ASD in SED‐S 4 showed higher scores in the domain *Peers* than those with ID only. Finally, results indicated an uneven distribution of ‘yes’ responses to the items, significant differences between the two groups in the number of yes‐responses to 27 of the 200 items, and a lower average of ‘yes’ answers from people with ID and ASD.

We found heterogeneous developmental profiles for people with ID and ASD. More specifically, people with ID and ASD showed particularly lower scores in the domains *Peers* and *Emotions* and particularly higher scores in the domains *Materials* and *Object* as compared to scores in the other domains. Similarly, prior research reported relatively lower mean scores for *Peers* and *Emotions* and relatively higher mean scores for *Object* among people with ID and ASD (Sappok, Heinrich, and Böhm 2020). Lower scores in the domain *Peers* and *Emotions* may not be surprising given that ASD symptomology include limitations in the ability to maintain and sustain peer relationships (ICD‐11; World Health Organization 2018). Furthermore, studies have highlighted challenges for individuals with ASD with understanding, recognizing and describing their own emotions (Berthoz and Hill 2005; Rieffe et al. 2007; Samson et al. 2012). Of note, the *Object* domain focuses on the extent to which a person has achieved the developmental milestone of object and person permanence. It has been suggested that this domain is more closely associated with cognitive abilities rather than emotional abilities (Sterkenburg et al. 2021), which may explain why this domain is less affected in people with ID and ASD. Furthermore, people with ID and ASD may tend to score higher in the domain *Materials* because the items, especially in the lower SED‐S levels, primarily focus on self‐directed behaviours, for example, individual engagement with objects and materials rather than social interactions.

Subgroup comparisons indicated lower mean scores for people with ID and ASD across all eight SED‐S domains. These findings underscore that people with ID and ASD experience more widespread challenges in ED compared with those with ID only. A previous study by Sappok, Heinrich, and Böhm (2020) also reported lower levels of ED in all eight SED‐S domains. However, when the authors stratified the results according to level of ID, people with mild/moderate ID and ASD exhibited lower levels of ED in three SED‐S domains and people with severe/profound ID displayed lower levels of ED in five SED‐S domains. The slight discrepancy in results may be due to the use of different statistical approaches and different levels of statistical power; we included a larger group of participants (*n* = 174 with ID and ASD, and 435 with ID only) than the study of Sappok, Heinrich, and Böhm (2020) (*n* = 102 with ID and ASD and 187 with ID only).

Follow‐up exploratory analyses were conducted to examine whether differences in domain scores remained or disappeared when groups were stratified according to SED‐S total score; few differences in ED were found. People with ID and ASD in SED‐S 2 showed lower scores in the domain *Affect* and people with ID and ASD in SED‐S 3 showed lower scores in the domains *Affect*, *Communication* and *Peers* than their respective counterparts with ID only. Surprisingly, people with ID and ASD in SED‐S 4 showed higher scores in the domain *Peers* compared to those with ID only in this respective stage. These findings may suggest that people with ID and ASD are more developed in their interactions with peers by the point they reach SED‐4. However, results should be interpreted with caution due to the small number of participants with ID and ASD assigned to SED‐S 4. Also note that the number of yes‐responses did not differ between individuals with ID and ASD and those with ID only for the domain *Peers* in SED‐S 4. Future studies are needed to replicate these exploratory findings.

While a recent study was the first to examine the psychometric properties of the items on the SED‐S (Hermann et al. 2024), this study was the first to examine differences in ED between individuals with ID and ASD and those with ID alone on item level. Three important findings emerged: (1) there was a heterogeneous distribution of yes‐responses, (2) there were significant differences between the groups in the number of yes‐responses provided to 27 of the 200 items and (3) people with ID and ASD scored on average fewer yes‐answers than those with ID only. Similar to the domains, some items showed considerable overlap with autism symptomology, making it unsurprising that they received fewer ‘yes’ responses from individuals with ID and ASD—for example, ‘Is able to name own basic feelings’ (SED‐S 3, *Emotion* domain, item 4)—or more ‘yes’ responses, for example, ‘Displays auto‐aggressive behaviour’ (SED‐S 1, domain *Affect*, item 5). Importantly, results of the item analyses provide a valuable first step towards refining treatment programs for individuals with ID and ASD. For example, items such as ‘Body‐focused attention leads to pleasurable interactions’ (SED‐S 2, domain *Body*, item 5) and ‘Wants to be the center of attention’ (SED‐S 3, domain *Emotion*, item 5) appear less applicable to individuals with ID and ASD. By integrating these insights, clinicians may develop more personalized intervention strategies.

Strengths of the study include the matching of individuals with ID and ASD to a group of individuals with ID only, the relatively large sample size, the comprehensive analyses of differences in ED between people with ID and ASD and those with ID only, and the adherence to the preregistration (supplemented by post hoc analyses of domain scores by SED‐S level). However, there are some limitations that should be noted. First, we found a relatively high rate of psychiatric comorbidity in our sample, which may have impacted the results. Second, it remains unclear whether the interviewees faced difficulties with the assessment of behaviour in people with ASD. Future studies may benefit from combining quantitative results with qualitative evaluations, e.g. rating by experts. Third, a relatively small number of participants were assigned to SED‐S 4 and SED‐S 5, making it difficult to draw firm conclusions. Future studies with more participants assigned to the higher SED‐stages are therefore needed. Finally, diagnoses of ID and ASD were based on ICD‐10 criteria. As this was a European multicentre study, there was no standardized procedure for assigning a diagnosis of ASD. Nevertheless, the diagnostic criteria were consistently applied making it unlikely that this has affected the results.

## 5 Conclusion

This preregistered study aimed to better understand differences in ED between people with ID and ASD and those with ID only. We add a piece to the ED puzzle by demonstrating that individuals with ID and ASD showed lower levels of ED overall and in all eight SED‐S domains than individuals with ID only. When groups were stratified according to SED‐S total scores, few differences in ED were found. Results of the item analyses provided insight into which items are particularly relevant or indicative for individuals with ASD. Although this study was largely exploratory and warrants replication, we provide an important next step towards a better understanding of the emotional needs and behaviours of people with ID and those with an additional ASD diagnosis.

## Ethics Statement

Prior to data collection, all participating organizations received ethical approval from an independent review board.

## Conflicts of Interest

Tanja Sappok received royalties from Hogrefe and Kohlhammer for the publication of the SED‐S manual and other book projects. The other authors declare no conflicts of interest.

## Supporting information


**Table S1.** Results of the nonparametric multiple comparison test for repeated measures for the ID/ASD group.
**Table S2.** Yes‐responses to SED‐S 1 items for people with ID/ASD and ID.
**Table S3.** Yes‐responses to SED‐S 2 items for people with ID/ASD and ID.
**Table S4.** Yes‐responses to SED‐S 3 items for people with ID/ASD and ID.
**Table S5.** Yes‐responses to SED‐S 4 items for people with ID/ASD and ID.
**Table S6.** Yes‐responses to SED‐S 5 items for people with ID/ASD and ID.

## Data Availability

Data is available upon reasonable request.
