# Supplementary material for: Comparing Emotional Development in Persons With Intellectual Disability With and Without Autism Spectrum Disorder
Source: J Intellect Disabil Res. 2025 May 14;69(10):1143–53. doi: 10.1111/jir.13251 (PMC12576381; doi:10.1111/jir.13251)
Supplement: Supplementary file 1 — Table S1. Results of the nonparametric multiple comparison test for repeated measures for the ID/ASD group. Table S2. Yes‐responses to SED‐S 1 items for people with ID/ASD and ID. Table S3. Yes‐responses to SED‐S 2 items for people with ID/ASD and ID. Table S4. Yes‐responses to SED‐S 3 items for people with ID/ASD and ID. Table S5. Yes‐responses to SED‐S 4 items for people with ID/ASD and ID. Table S6. Yes‐responses to SED‐S 5 items for people with ID/ASD and ID. [file JIR-69-1143-s001.docx]

**Supplementary Materials**

**Supplementary Table 1**. Results of the nonparametric multiple comparison test for repeated measures for the ID/ASD group.

| Comparison | Estimator | *p* value |
| --- | --- | --- |
| 2 vs.1 | -.0484 | .0052 |
| 3 vs.1 | .0781 | .0001 |
| 4 vs.1 | -.153 | <.0001 |
| 5 vs.1 | -.196 | <.0001 |
| 6 vs.1 | .0028 | .8774 |
| 7 vs.1 | -.0505 | .0065 |
| 8 vs.1 | -.1476 | <.0001 |
| 3 vs.2 | .1265 | <.0001 |
| 4 vs.2 | -.1046 | <.0001 |
| 5 vs.2 | -.1476 | <.0001 |
| 6 vs.2 | .0512 | .0058 |
| 7 vs.2 | -.0021 | .9049 |
| 8 vs.2 | -.0992 | <.0001 |
| 4 vs.3 | -.2311 | <.0001 |
| 5 vs.3 | -.2741 | <.0001 |
| 6 vs.3 | -.0753 | .0002 |
| 7 vs.3 | -.1286 | <.0001 |
| 8 vs.3 | -.2257 | <.0001 |
| 5 vs.4 | -.043 | .0109 |
| 6 vs.4 | .1558 | <.0001 |
| 7 vs.4 | .1025 | <.0001 |
| 8 vs.4 | .0054 | .7407 |
| 6 vs.5 | .1988 | <.0001 |
| 7 vs.5 | .1455 | <.0001 |
| 8 vs.5 | .0484 | .0062 |
| 7 vs.6 | -.0533 | .0016 |
| 8 vs.6 | -.1504 | <.0001 |
| 8 vs.7 | -.0971 | <.0001 |

*Note*. The above calculations correspond to the following numbering: *Body* (1), *Others* (2), *Object* (3), *Emotions* (4), *Peers* (5), *Material* (6), *Communication* (7), *Affect* (8). The estimator < 0 indicates a tendency to lower values and the estimator > 0 indicates a tendency to higher values. For example, domain 5 (Peers) tends to have lower values than domain 1 (Body) (estimate -.196).

**Supplementary Table 2**. Yes-responses to SED-S 1 items for people with ID/ASD and ID

| SED-S 1 items | Domain and item number | Overall | ID/ASD *n* (%) | ID group *n* (%) | *p*-value |
| --- | --- | --- | --- | --- | --- |
| - | - | 70 (100) | 48 (100) | 22 (100) | - |
| Emotional states are largely determined by basic physical sensations and needs (hunger, thirst, pain, fatigue, cold) | Body 1_1 | 43 | 31 (65) | 12 (55) | .423 |
| Only feels safe and secure in a familiar environment, i.e. when surrounded by familiar faces and stimuli (e.g. touch, smells, sounds, etc.) | Body 1_2 | 37 | 26 (54) | 11 (50) | .746 |
| Passively enjoys sensory stimulation (e.g. being cared or touched) | Body 1_3 | 41 | 26 (54) | 15 (68) | .269 |
| Explores his/her body randomly by touching, grasping, (thumb) sucking etc. | Body 1_4 | 24 | 18 (38) | 6 (27) | .403 |
| Engages with his/her body by means of repetitive movements (flapping arms, rocking back and forth etc.) and vocalizations | Body 1_5 | 49 | 33 (69) | 16 (73) | .736 |
|  |  |  |  |  |  |
| Social interaction mainly arises when basic needs (e.g. food, hygiene, touch) are being met | Others 1_1 | 53 | 37 (77) | 16 (73) | .693 |
| Is soothed by physical contact with significant others (e.g. hugging, rocking) | Others 1_2 | 30 | 18 (38) | 12 (55) | .181 |
| Primarily interacts with his/her environment via the proximal senses (e.g. touch, smell and taste) | Others 1_3 | 32 | 23 (48) | 9 (41) | .585 |
| Enjoys feeling the spatial boundaries of his/her body through intensive physical contact with significant others  (e.g. being held, caresses, massages, rocking, tickling) | Others 1_4 | 30 | 18 (38) | 12 (55) | .181 |
| Contact is more difficult when there is a lot of commotion or noise (sensory overload) | Others 1_5 | 45 | 32 (67) | 13 (59) | .539 |
|  |  |  |  |  |  |
| Emotional states are primarily determined by the immediate situation | Object 1_1 | 55 | 38 (79) | 17 (77) | .858 |
| The environment is perceived solely through the senses | Object 1_2 | 44 | 29 (60) | 15 (68) | .533 |
| Persons or object that cannot be perceived using any sense no longer exist | Object 1_3 | 21 | 14 (29) | 7 (32) | .822 |
| Does not look for hidden/lost objects (“out of sight, out of mind”) | Object 1_4 | 31 | 20 (42) | 11 (50) | .515 |
| Lives in the “here and now” | Object 1_5 | 56 | 40 (83) | 16 (73) | .303 |
|  |  |  |  |  |  |
| Emotional states are expressed with the entire body | Emotion 1_1 | 53 | 39 (81) | 14 (64) | .111 |
| The expression of emotions changes rapidly in response to stimuli | Emotion 1_2 | 39 | 23 (48) | 16 (73) | .052 |
| Emotions are expressed intensely with no variation in degree | Emotion 1_3 | 38 | 28 (58) | 10 (45) | .315 |
| Reacts to sensory overload by retreating or shutting out stimuli (e.g. covering his/her ears) | Emotion 1_4 | 37 | 25 (52) | 12 (55) | .848 |
| Responds to aversive stimuli with a mix of tension, anxiety and anger | Emotion 1_5 | 45 | 33 (69) | 12 (55) | .250 |
|  |  |  |  |  |  |
| Shows no interest in peers | **Peers 1_1** | **48** | **38 (79)** | **10 (45)** | **.005** |
| Sees others as objects rather than beings who act and react | Peers 1_2 | 40 | 25 (52) | 15 (68) | .206 |
| Interacts with peers according to fixed patterns | Peers 1_3 | 28 | 19 (40) | 9 (41) | .916 |
| Actions elicit responses from peers | Peers 1_4 | 33 | 20 (42) | 13 (59) | .175 |
| Forced contact with peers results in noticeable tension | Peers 1_5 | 31 | 23 (48) | 8 (36) | .366 |
|  |  |  |  |  |  |
| Is mainly preoccupied with his/her own body | Material 1_1 | 43 | 27 (56) | 16 (73) | .189 |
| Only engages with materials and objects within immediate reach | Material 1_2 | 26 | 15 (31) | 11 (50) | .132 |
| Primarily engages in repetitive activities that stimulate the senses (e.g. rocking, making sounds, touching) | Material 1_3 | 53 | 36 (75) | 17 (77) | .837 |
| Enjoys handling and exploring amorphous materials such as water, sand or soap bubbles | Material 1_4 | 34 | 25 (52) | 9 (41) | .385 |
| Explores things by feeling, licking and tasting them | Material 1_5 | 26 | 19 (40) | 7 (32) | .533 |
|  |  |  |  |  |  |
| Undirected expression of inner states using the entire body | Communication 1_1 | 47 | 34 (71) | 13 (59) | .332 |
| Verbal communication and comprehension are lacking | Communication 1_2 | 22 | 16 (33) | 6 (27) | .612 |
| Conveys emotional states by crying, shrieking, clinging to caregivers, pacing back and forth | Communication 1_3 | 46 | 31 (65) | 15 (68) | .768 |
| Imitates facial expressions, sounds and words | Communication 1_4 | 10 | 8 (17) | 2 (9) | .400 |
| Keeps repeating the same words and sounds | Communication 1_5 | 45 | 28 (58) | 17 (77) | .125 |
|  |  |  |  |  |  |
| Tension is relieved by (active or passive) movement and physical touch | Affect 1_1 | 50 | 35 (73) | 15 (68) | .684 |
| Responds to physical sensations and external stimuli with tension | Affect 1_2 | 56 | 38 (79) | 18 (82) | .797 |
| Uses repetitive movements (e.g. rocking, flapping arms, hitting etc.) as a means of self-regulation | Affect 1_3 | 46 | 34 (71) | 12 (55) | .183 |
| Has no control over his/her impulses | Affect 1_4 | 49 | 35 (73) | 14 (64) | .432 |
| Displays auto-aggressive behavior | **Affect 1_5** | **57** | **43 (90)** | **14 (64)** | **.010** |

*Note.* The items with significant differences are marked in bold.

**Supplementary Table 3**. Yes-responses to SED-S 2 items for people with ID/ASD and ID

| SED-S 2 items | Domain and item number | Overall | ID/ASD *n* (%) | ID group *n* (%) | *p*-value |
| --- | --- | --- | --- | --- | --- |
| - | - | 105 (100) | 61 (100) | 44 (100) | *-* |
| Uses mouth as well as hands to explore his/her environment | Body 2_1 | 26 | 13 (21) | 13 (30) | .335 |
| Uses his/her body as an instrument to explore the immediate environment (e.g. switching the light on and off repeatedly etc.) | **Body 2_2** | **52** | **25 (41)** | **27 (61)** | **.039** |
| Smears feces and body fluids (saliva, blood, sperm) | Body 2_3 | 31 | 14 (23) | 17 (39) | .082 |
| Uses his/her body to manipulate objects in the immediate environment | Body 2_4 | 39 | 20 (33) | 19 (43) | .277 |
| Body-focused attention (e.g. during bathing, brushing hair) leads to pleasurable interactions | **Body 2_5** | **49** | **22 (36)** | **27 (61)** | **.010** |
|  |  |  |  |  |  |
| Sense of wellbeing is dependent on the presence of significant others | Others 2_1 | 59 | 37 (61) | 22 (50) | .278 |
| Shows a preference for contact with significant others | Others 2_2 | 86 | 50 (82) | 36 (82) | .984 |
| Stays close to significant others in unfamiliar surroundings | Others 2_3 | 69 | 42 (69) | 27 (61) | .425 |
| Protests when contact with significant others is lost or ended | Others 2_4 | 36 | 21 (34) | 15 (34) | .972 |
| Actively seeks physical proximity to significant others and follows them like a shadow | Others 2_5 | 61 | 31 (51) | 30 (68) | .075 |
|  |  |  |  |  |  |
| Is tense and upset when separated from significant others | Object 2_1 | 33 | 21 (34) | 12 (27) | .436 |
| Significant others provide a sense of security in unfamiliar situations | Object 2_2 | 81 | 31 (51) | 30 (68) | .063 |
| Briefly looks for things that have disappeared from sight | Object 2_3 | 42 | 24 (39) | 18 (41) | .872 |
| Enjoys playing peek-a-boo and hide-and-seek | **Object 2_4** | **23** | **8 (13)** | **15 (34)** | **.010** |
| Frequently seeks verbal reassurance from significant others | Object 2_5 | 44 | 23 (38) | 21 (48) | .304 |
|  |  |  |  |  |  |
| Reacts negatively when contact with significant others is lacking | Emotion 2_1 | 51 | 32 (52) | 19 (43) | .348 |
| Enjoys activities with significant others | **Emotion 2_2** | **81** | **42 (69)** | **39 (89)** | **.017** |
| Dislikes being alone and seeks contact with (significant) others | Emotion 2_3 | 64 | 33 (54) | 31 (70) | .090 |
| Emotional states can be influenced by attention from caregivers | **Emotion 2_4** | **80** | **42 (69)** | **38 (86)** | **.038** |
| Is noticeably tense in unfamiliar/confusing social situations | Emotion 2_5 | 65 | 37 (61) | 28 (64) | .756 |
|  |  |  |  |  |  |
| Engages in the same activities as peers, but independently of them (i.e. “parallel play” – playing side by side rather than with each other) | Peers 2_1 | 54 | 28 (46) | 26 (59) | .182 |
| Is curious about peers and interacts with them briefly | **Peers 2_2** | **47** | **22 (36)** | **25 (57)** | **.035** |
| Tries to find out more about peers by watching, listening and/or touching | Peers 2_3 | 64 | 33 (54) | 31 (70) | .090 |
| Imitates peers when interacting with others | Peers 2_4 | 18 | 8 (13) | 10 (23) | .197 |
| Engages with peers when authority figures are present | **Peers 2_5** | **34** | **15 (25)** | **19 (43)** | **.045** |
|  |  |  |  |  |  |
| Manipulates objects repetitively for sustained periods of time (e.g. putting them into/taking them out of boxes, moving them back and forth, throwing them etc,) | Material 2_1 | 46 | 27 (44) | 19 (43) | .912 |
| Interacting with significant others is the main focus of activities | Material 2_2 | 45 | 22 (36) | 23 (52) | .098 |
| Enjoys interactions that involve repeatedly throwing, dropping and handing objects back and forth | **Material 2_3** | **21** | **7 (11)** | **14 (32)** | **.010** |
| Explores/examines materials and objects by kneading, hitting and shaking them | Material 2_4 | 29 | 16 (26) | 13 (30) | .708 |
| Reaches for things he/she can see or hear | **Material 2_5** | **57** | **27 (44)** | **30 (68)** | **.015** |
|  |  |  |  |  |  |
| Frequently clings to significant others | Communication 2_1 | 43 | 22 (36) | 21 (48) | .231 |
| Seeks constant physical and verbal contact with significant others by remaining close by their side | Communication 2_2 | 52 | 27 (44) | 25 (57) | .204 |
| Points at things to get others’ attention | Communication 2_3 | 59 | 33 (54) | 26 (59) | .611 |
| Repeats sounds, words and short sentences he/she hears | Communication 2_4 | 43 | 24 (39) | 19 (43) | .693 |
| Uses his/her entire body to get his/her message across | Communication 2_5 | 77 | 42 (69) | 35 (80) | .222 |
|  |  |  |  |  |  |
| Negative emotions are regulated when the cause of stress is eliminated | Affect 2_1 | 78 | 44 (72) | 34 (77) | .552 |
| Turns to significant others for protection and consolation | Affect 2_2 | 56 | 28 (46) | 28 (64) | .072 |
| Is able to control (aggressive) impulses if significant others are nearby and ready to intervene if necessary | Affect 2_3 | 70 | 41 (67) | 29 (66) | .889 |
| Is upset and angry when significant others leave | Affect 2_4 | 25 | 14 (23) | 11 (25) | .808 |
| Frustration leads to anger that can be manifested in aggressive behavior towards significant others, physical restlessness, screaming, hitting or throwing objects | Affect 2_5 | 69 | 40 (66) | 29 (66) | .972 |

*Note.* The items with significant differences are marked in bold.

**Supplementary Table 4**. Yes-responses to SED-S 3 items for people with ID/ASD and ID

| **SED-S 3 items** | **Domain and item number** | **Overall** | **ID/ASD *n* (%)** | **ID group *n* (%)** | ***p*-value** |
| --- | --- | --- | --- | --- | --- |
| - | - | 107 (100) | 53 (100) | 54 (100) | - |
| Wants to do everything him-/herself (e.g. personal hygiene, meals, etc.) | Body 3_1 | 63 | 28 (53) | 35 (65) | .208 |
| **Seeks help from others or utilizes objects to overcome physical limitations (uses a chair to reach the cookie jar, for example)** | **Body 3_2** | **87** | **37 (70)** | **50 (93)** | **.003** |
| Tries to assert his/her will by means of provocative behavior (e.g. stripping off clothes, stomping, flinging him-/herself on the ground) | Body 3_3 | 56 | 26 (49) | 30 (56) | .501 |
| **Uses language (possibly supported by gestures) to communicate** | **Body 3_4** | **99** | **46 (87)** | **53 (98)** | **.026** |
| Goes to the toilet on his/her own, but needs help with hygiene | Body 3_5 | 55 | 24 (45) | 31 (57) | .210 |
|  |  |  |  |  |  |
| Insists on getting his/her way | Others 3_1 | 92 | 45 (85) | 47 (87) | .751 |
| Wants attention and tests limits at the same time. | Others 3_2 | 71 | 35 (66) | 36 (67) | .945 |
| Is open to alternative suggestions | Others 3_3 | 83 | 39 (74) | 44 (81) | .328 |
| Only obeys rules when authority figures are present | Others 3_4 | 46 | 24 (45) | 22 (41) | .635 |
| Persistently says “No!” in order to assert his/her will | Others 3_5 | 69 | 34 (64) | 35 (65) | .943 |
|  |  |  |  |  |  |
| Is upset at loss of transitional objects (i.e. emotionally charged “comfort” objects) | Object 3_1 | 28 | 11 (21) | 17 (31) | .207 |
| Transitional/comfort objects provide a sense of security | Object 3_2 | 31 | 13 (25) | 18 (33) | .315 |
| Intentionally looks for things and people that can no longer be seen/heard | Object 3_3 | 80 | 38 (72) | 42 (78) | .469 |
| Emotional bonds are maintained over a distance (e.g. over the telephone) | Object 3_4 | 80 | 39 (74) | 41 (76) | .780 |
| Shows resentment after being separated from significant others for longer periods of time | Object 3_5 | 19 | 8 (15) | 11 (20) | .475 |
|  |  |  |  |  |  |
| Expresses anger when he/she doesn’t get his/her way | Emotion 3_1 | 79 | 35 (66) | 44 (81) | .069 |
| **Wants caregivers to him-/herself, shows jealousy** | **Emotion 3_2** | **43** | **16 (30)** | **27 (50)** | **.037** |
| Shows defiance towards significant others | Emotion 3_3 | 74 | 38 (72) | 36 (67) | .573 |
| **Is able to name own basic feelings (e.g. anger, sadness, fear, happiness)** | **Emotion 3_4** | **58** | **22 (42)** | **36 (67)** | **.009** |
| **Wants to be the center of attention** | **Emotion 3_5** | **46** | **16 (30)** | **30 (56)** | **.008** |
|  |  |  |  |  |  |
| **Tries to impose his/her will on peers** | **Peers 3_1** | **60** | **22 (42)** | **38 (70)** | **.003** |
| Is reluctant to share materials with peers | Peers 3_2 | 34 | 13 (25) | 21 (39) | .111 |
| **Shows no regard for what peers want** | **Peers 3_3** | **57** | **22 (42)** | **35 (65)** | **.016** |
| Is jealous of peers | Peers 3_4 | 37 | 14 (26) | 23 (43) | .079 |
| **Bosses peers around** | **Peers 3_5** | **47** | **16 (30)** | **31 (57)** | **.005** |
|  |  |  |  |  |  |
| Shares only reluctantly or at the request of significant others | Material 3_1 | 46 | 21 (40) | 25 (46) | .486 |
| Engages in activities requiring fine motor skills, such as cutting and pasting | Material 3_2 | 81 | 39 (74) | 42 (78) | .613 |
| The activity itself is more important than the end result (has no interest in keeping drawings once they’re finished, for example) | Material 3_3 | 57 | 30 (57) | 27 (50) | .494 |
| Frequently examines objects which may then fall apart | Material 3_4 | 18 | 7 (13) | 11 (20) | .322 |
| **Does activities in which imitation plays a role** | **Material 3_5** | **20** | **5 (9)** | **15 (28)** | **.015** |
|  |  |  |  |  |  |
| Often says ‘”no” to try to get his/her way | Communication 3_1 | 70 | 36 (68) | 34 (63) | .590 |
| Communication is self-centered | Communication 3_2 | 84 | 45 (85) | 39 (72) | .110 |
| Communication is mostly related to the immediate situation | Communication 3_3 | 53 | 28 (53) | 25 (46) | .499 |
| **Uses “bad” words to provoke reactions from others** | **Communication 3_4** | **31** | **10 (19)** | **21 (39)** | **.022** |
| Says things with no regard for the social context | Communication 3_5 | 64 | 31 (58) | 33 (61) | .782 |
|  |  |  |  |  |  |
| Responds with aggression when limits are imposed on his/her will | Affect 3_1 | 73 | 35 (66) | 38 (70) | .630 |
| Is more willing to cooperate when offered a choice between two alternatives | Affect 3_2 | 69 | 31 (58) | 38 (70) | .199 |
| **Frustration finds expression in physical restlessness, temper tantrums and stubbornly oppositional behavior** | **Affect 3_3** | **89** | **39 (74)** | **50 (93)** | **.009** |
| Frequently conveys protest using physical or verbal means | Affect 3_4 | 78 | 37 (70) | 41 (76) | .477 |
| **Is rarely able to talk about the causes and effects of his/her aggressive behavior** | **Affect 3_5** | **67** | **27 (51)** | **40 (74)** | **.013** |

*Note.* The items with significant differences are marked in bold.

**Supplementary Table 5**. Yes-responses to SED-S 4 items for people with ID/ASD and ID

| SED-S 4 items | Domain and item number | Overall | ID/ASD *n* (%) | ID group *n* (%) | *p*-value |
| --- | --- | --- | --- | --- | --- |
| - | - | 52 (100) | 8 (100) | 44 (100) | *-* |
| Seeks role models of the same gender to identify with (e.g. police officers, firefighters, pop stars, caregivers) | Body 4_1 | 24 | 2 (25) | 22 (50) | .192 |
| Has developed a gender identity | Body 4_2 | 51 | 8 (100) | 43 (98) | .667 |
| Shows a sense of shame/modesty (closes the door when using the toilet, for example) | Body 4_3 | 39 | 5 (62) | 34 (77) | .375 |
| Seeks to imitate role models in terms of appearance (e.g. by adopting hair and clothing styles, necklaces, bracelets etc.) | Body 4_4 | 18 | 2 (25) | 16 (36) | .534 |
| Wants to choose clothing according to personal taste, regardless of the weather or occasion | Body 4_5 | 14 | 0 (0) | 14 (32) | .062 |
|  |  |  |  |  |  |
| Solicits the opinion and seeks the approval of significant others | Others 4_1 | 35 | 5 (62) | 30 (68) | .753 |
| Wants to assume the role of authority figures (“deputy caregiver”) | Others 4_2 | 24 | 3 (38) | 21 (48) | .594 |
| Makes decisions on his/her own and is aware of the immediate consequences (when crossing the street, for example) | Others 4_3 | 35 | 5 (62) | 30 (68) | .753 |
| Identifies with role models and attempts to imitate their characteristic behavior | Others 4_4 | 16 | 1 (12) | 15 (34) | .224 |
| Wants to be perceived and treated in accordance with his/her gender | Others 4_5 | 37 | 4 (50) | 33 (75) | .151 |
|  |  |  |  |  |  |
| Can manage transitions between familiar social contexts (e.g. residential and work settings) | Object 4_1 | 45 | 6 (75) | 39 (89) | .299 |
| Is able to part with transitional objects in familiar environments | Object 4_2 | 12 | 1 (12) | 11 (25) | .440 |
| Authority figures provide reassurance in unfamiliar situations | Object 4_3 | 46 | 7 (88) | 39 (89) | .926 |
| Can engage in familiar activities in unfamiliar environments | Object 4_4 | 42 | 5 (62) | 37 (84) | .154 |
| Is insecure in unfamiliar environments when no authority figures are present | Object 4_5 | 33 | 5 (62) | 28 (64) | .951 |
|  |  |  |  |  |  |
| Is afraid of making mistakes or doing something wrong | Emotion 4_1 | 30 | 5 (62) | 25 (57) | .765 |
| Is able to empathize with others (tries to console them, for example) | Emotion 4_2 | 36 | 4 (50) | 32 (73) | .200 |
| Shows feelings of guilt | Emotion 4_3 | 31 | 5 (62) | 26 (59) | .857 |
| Shows a sense of shame/modesty (facial expression) | Emotion 4_4 | 36 | 4 (50) | 32 (73) | .200 |
| Is worried he/she won’t be able to handle assigned tasks or responsibilities | Emotion 4_5 | 23 | 5 (62) | 18 (41) | .258 |
|  |  |  |  |  |  |
| Has regular, sustained contact with peers | Peers 4_1 | 34 | 6 (75) | 28 (64) | .534 |
| Makes friends with certain peers | Peers 4_2 | 39 | 7 (88) | 32 (73) | .375 |
| Imitates caregivers when interacting with peers | Peers 4_3 | 17 | 3 (38) | 14 (32) | .753 |
| Frequently enjoys activities with peers for sustained periods, but is still primarily focused on what he/she wants | Peers 4_4 | 35 | 4 (50) | 31 (70) | .257 |
| Voluntarily shares material with peers | Peers 4_5 | 43 | 7 (88) | 36 (82) | .696 |
|  |  |  |  |  |  |
| Approaches peers by play and shared activities | **Material 4_1** | **34** | **1 (12)** | **33 (75)** | **.001** |
| Likes games involving imagination (playing “pretend” and make-believe) | Material 4_2 | 14 | 2 (25) | 12 (27) | .894 |
| Engages in creative activities such as painting and drawing | Material 4_3 | 22 | 5 (62) | 17 (39) | .209 |
| Shows imagination and creativity by varying colours, shapes etc. when working with materials | Material 4_4 | 25 | 4 (50) | 21 (48) | .906 |
| Follows simple rules (like waiting his/her turn) when playing games | **Material 4_5** | **51** | **7 (88)** | **44 (100)** | **.018** |
|  |  |  |  |  |  |
| Continuously asks “why” questions | Communication 4_1 | 19 | 2 (25) | 17 (39) | .461 |
| Asks questions and contributes information in conversations relating to his/her personal experience and daily life | Communication 4_2 | 36 | 6 (75) | 30 (68) | .701 |
| Describes his/her emotions in simple terms | Communication 4_3 | 44 | 6 (75) | 38 (86) | .413 |
| Can explain in simple terms why he/she is upset, happy, scared, jealous, proud etc. | Communication 4_4 | 41 | 5 (62) | 36 (82) | .218 |
| Communication is directed at peers as well as significant others | Communication 4_5 | 49 | 8 (100) | 41 (93) | .447 |
|  |  |  |  |  |  |
| Expresses regret and wants to make amends | Affect 4_1 | 32 | 4 (50) | 28 (64) | .466 |
| Talks about what he/she regards as good or acceptable behavior and gives reasons | Affect 4_2 | 17 | 4 (50) | 13 (30) | .257 |
| Can delay gratification of his/her own wishes for the sake of a reward | Affect 4_3 | 28 | 2 (25) | 26 (59) | .075 |
| Is able to talk about the causes and effects of his/her aggressive behavior with support from significant others | Affect 4_4 | 32 | 4 (50) | 28 (64) | .466 |
| Is a “sore loser” and refuses to continue playing after losing a game | Affect 4_5 | 8 | 0 (0) | 8 (18) | .190 |

*Note.* The items with significant differences are marked in bold.

**Supplementary Table 6**. Yes-responses to SED-S 5 items for people with ID/ASD and ID

| SED-S 5 items | Domain and item number | Overall | ID/ASD *n* (%) | ID group *n* (%) | *p*-value |
| --- | --- | --- | --- | --- | --- |
| - |  | 14 (100) | 4 (100) | 10 (100) | **-** |
| Seeks to assert his/her position in the peer group by means of his/her appearance | Body 5_1 | 6 | 1 (25) | 5 (50) | .393 |
| Is eager to demonstrate physical prowess and compete with others | Body 5_2 | 7 | 1 (25) | 6 (60) | .237 |
| Shows modesty/a sense of shame in respect to sexuality | Body 5_3 | 13 | 4 (100) | 9 (90) | .512 |
| Is able to assess his/her physical abilities accurately | Body 5_4 | 12 | 3 (75) | 9 (90) | .469 |
| Is concerned with his/her appearance and increasingly able to gauge how he/she is perceived by others realistically | Body 5_5 | 7 | 2 (50) | 5 (50) | 1.00 |
|  |  |  |  |  |  |
| Is eager to compete with significant others | Others 5_1 | 7 | 2 (50) | 5 (50) | 1.00 |
| Conforms to social norms and rules even when no authority figures are present | Others 5_2 | 12 | 3 (75) | 9 (90) | .469 |
| Models behavior on peers more than on authority figures | Others 5_3 | 6 | 2 (50) | 4 (40) | .733 |
| Looks to individuals outside his/her immediate everyday environment (e.g. athletic coaches etc.) for guidance | Others 5_4 | 5 | 1 (25) | 4 (40) | .597 |
| Seeks to gain approval by taking on responsibilities or tasks and pointing accomplishments out to significant others (“look what I did”) | Others 5_5 | 11 | 3 (75) | 8 (80) | .837 |
|  |  |  |  |  |  |
| Initiates social activities on his/her own | Object 5_1 | 13 | 4 (100) | 9 (90) | .512 |
| Explores unfamiliar environments of his/her own accord | Object 5_2 | 12 | 3 (75) | 9 (90) | .469 |
| Adapts to changing situational demands | Object 5_3 | 14 | 4 (100) | 10 (100) | n.a. |
| Pursues interests beyond his/her familiar environment | Object 5_4 | 13 | 3 (75) | 10 (100) | .101 |
| Applies familiar behavior in new contexts | Object 5_5 | 14 | 4 (100) | 10 (100) | n.a. |
|  |  |  |  |  |  |
| Is worried about not being accepted by peers | Emotion 5_1 | 9 | 2 (50) | 7 (70) | .480 |
| Is concerned about his/her appearance | Emotion 5_2 | 8 | 1 (25) | 7 (70) | .124 |
| Strives to win the approval of peers | Emotion 5_3 | 9 | 2 (50) | 7 (70) | .480 |
| Is concerned about violating social conventions and rules | Emotion 5_4 | 7 | 1 (25) | 6 (60) | .237 |
| Reflects on and regulates his/her emotions | Emotion 5_5 | 10 | 4 (100) | 6 (60) | .134 |
|  |  |  |  |  |  |
| Maintains close friendships with peers | Peers 5_1 | 9 | 2 (50) | 7 (70) | .480 |
| Is competitive with peers | Peers 5_2 | 5 | 0 (0) | 5 (50) | .078 |
| Wants to “belong” and be popular with peers | Peers 5_3 | 11 | 3 (75) | 8 (80) | .837 |
| Shows loyalty to friends | **Peers 5_4** | **12** | **2 (50)** | **10 (100)** | **.016** |
| Consults with peers to find solutions to conflicts/problems | **Peers 5_5** | **8** | **0 (0)** | **8 (80)** | **.006** |
|  |  |  |  |  |  |
| Can share materials, tasks and responsibilities in activities with peers | Material 5_1 | 14 | 4 (100) | 10 (100) | n.a. |
| The primary aim of activities is achieving a good result (saves only “nice” drawings, for example) | Material 5_2 | 13 | 3 (75) | 10 (100) | .101 |
| Receiving recognition for the final product/result is important | Material 5_3 | 10 | 3 (75) | 7 (70) | .852 |
| Works with others towards a common goal, e.g. in team sports and group activities | Material 5_4 | 9 | 2 (50) | 7 (70) | .480 |
| Participates in group activities of a competitive nature, such as races, bowling, etc. | Material 5_5 | 12 | 3 (75) | 9 (90) | .469 |
|  |  |  |  |  |  |
| Asks questions about cause and effect and how things work | Communication 5_1 | 9 | 2 (50) | 7 (70) | .480 |
| Communicates with peers and enjoys exchanges with them | Communication 5_2 | 13 | 4 (100) | 9 (90) | .512 |
| Uses objective/factual arguments to support his/her opinion in discussions | Communication 5_3 | 10 | 2 (50) | 8 (80) | .262 |
| Talks about his/her feelings | Communication 5_4 | 11 | 4 (100) | 7 (70) | .217 |
| Talks about his/her strengths and weaknesses | Communication 5_5 | 9 | 2 (50) | 7 (70) | .480 |
|  |  |  |  |  |  |
| Aggression is primarily expressed using verbal means | Affect 5_1 | 13 | 4 (100) | 9 (90) | .512 |
| Is able to regulate his/her emotions by talking about his/her feelings and needs and seeking support from others in the group | Affect 5_2 | 4 | 1 (25) | 3 (30) | .852 |
| Reflects on what did or didn’t go well without prompting from others | Affect 5_3 | 10 | 2 (50) | 8 (80) | .262 |
| Responds with aggression when he/she feels threatened, slighted or extremely frustrated | Affect 5_4 | 7 | 2 (50) | 5 (50) | 1.00 |
| Displays anger or aggression in competitive situations | Affect 5_5 | 1 | 0 (0) | 1 (10) | .512 |

*Note.* The items with significant differences are marked in bold.
